# Chemical and biophysical characterization of novel potassium channel blocker 3-fluoro-5-methylpyridin-4-amine

**DOI:** 10.1038/s41598-024-61465-w

**Published:** 2024-05-15

**Authors:** Yang Sun, Sofia Rodríguez-Rangel, Lauren L. Zhang, Jorge E. Sánchez-Rodríguez, Pedro Brugarolas

**Affiliations:** 1 Department of Radiology, Massachusetts General Hospital and Harvard Medical School, Boston, MA 02114 USA; 2 Departamento de Física, Universidad de Guadalajara, Guadalajara, Jalisco 44430 México

**Keywords:** Drug discovery, Physiology, Biomarkers

## Abstract

4-aminopyridine (4AP) is a potassium (K^+^) channel blocker used clinically to improve walking in people with multiple sclerosis (MS). 4AP binds to exposed K^+^ channels in demyelinated axons, reducing the leakage of intracellular K^+^ and enhancing impulse conduction. Multiple derivatives of 4AP capable of blocking K^+^ channels have been reported including three radiolabeled with positron emitting isotopes for imaging demyelinated lesions using positron emission tomography (PET). However, there remains a demand for novel molecules with suitable physicochemical properties and binding affinity that can potentially be radiolabeled and used as PET radiotracers. In this study, we introduce 3-fluoro-5-methylpyridin-4-amine (5Me3F4AP) as a novel trisubstituted K^+^ channel blocker with potential application in PET. 5Me3F4AP has comparable potency to 4AP and the PET tracer 3-fluoro-4-aminopyridine (3F4AP). Compared to 3F4AP, 5Me3F4AP exhibits comparable basicity (p*K*_a_ = 7.46 ± 0.01 vs. 7.37 ± 0.07, P-value = 0.08), greater lipophilicity (logD = 0.664 ± 0.005 vs*.* 0.414 ± 0.002, P-value < 0.0001) and higher permeability to an artificial brain membrane (*P*_*e*_ = 88.1 ± 18.3 vs*.* 31.1 ± 2.9 nm/s, P-value = 0.03). 5Me3F4AP is also more stable towards oxidation in vitro by the cytochrome P450 enzyme CYP2E1 (*IC*_*50*_ = 36.2 ± 2.5 vs*.* 15.4 ± 5.1, P-value = 0.0003); the enzyme responsible for the metabolism of 4AP and 3F4AP. Taken together, 5Me3F4AP has promising properties as a candidate for PET imaging warranting additional investigation.

## Introduction

4-aminopyridine (4AP) is a potassium channel blocker commonly used in the symptomatic treatment of multiple sclerosis (MS)^1–3^. Its mechanism of action involves binding to voltage-gated K^+^ (K_v_) channels exposed due to demyelination, reducing the aberrant efflux of K^+^ ions and enhancing axonal conduction^4–13^. Additionally, 4AP has demonstrated potential clinical utility for spinal cord injury^14–19^, traumatic brain injury^20^, and other diseases involving demyelination^21^. Based on the mechanism of action of 4AP, it has been proposed that upregulated K^+^ channels in demyelinated axons could be targeted for imaging demyelination using positron emission tomography (PET)^22,23^. Thus, a radiofluorinated derivative of 4AP, [^18^F]3-fluoro-4-aminopyridine ([^18^F]3F4AP), was synthesized and evaluated for imaging demyelination^22,24,25^. In those studies, [^18^F]3F4AP displayed high sensitivity in detecting demyelinated lesions in rodent models of MS^22^ and non-human primates^26^. Furthermore, [^18^F]3F4AP has shown acceptable radiation dosimetry in healthy human volunteers^27^ and it is currently undergoing evaluation in MS patients^28^ (ClinicalTrials.gov identifier: NCT04699747). Furthermore, since PET uses tracer doses pharmacological toxicity of these compounds is not a concern. Nevertheless, in humans [^18^F]3F4AP has shown lower metabolic stability (< 50% plasma parent fraction (PPF) remaining, 30 min post injection^27^) than in monkeys (> 90% PPF 2 h post injection^26^), which resulted in lower brain uptake in humans than expected from monkey studies^29^. This reduction in metabolic stability was found to arise from the inhibitory effect of isoflurane on the metabolism of [^18^F]3F4AP as evidenced by the fact that awake mice metabolize the tracer faster than anesthetized mice, with 17.7 ± 1.7 PPF (n = 11) *vs* 74.8 ± 1.6 PPF (n = 10) respectively 35 min post-injection^29^. Additional studies indicate that [^18^F]3F4AP is oxidized at the 5-position to 5-hydroxy-3F4AP by the cytochrome P450 enzyme CYP2E1^30^, which prompted us to look for more stable derivatives with suitable binding affinity and brain permeability.

**Figure 1:**
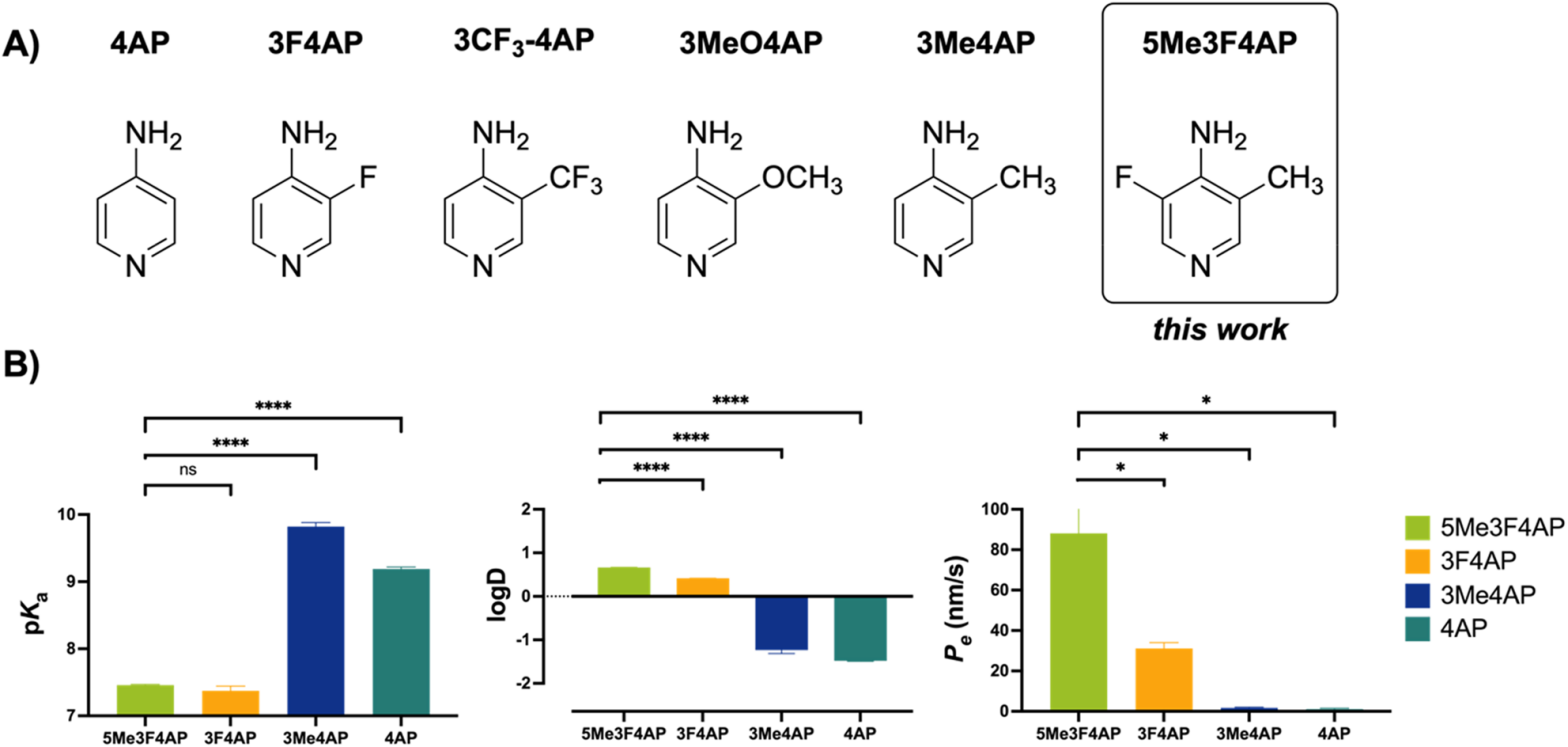
(**A**) Structures of 4AP, its derivatives explored for PET imaging, and 5Me3F4AP. (**B**) Comparison of p*K*_a_ (n = 4), logD (n = 4) and permeability (*Pe*, n = 3) to an artificial membrane between 5Me3F4AP, 3F4AP, 3Me4AP and 4AP. *P ≤ 0.05; **P ≤ 0.01, ***P ≤ 0.001, ****P ≤ 0.0001.

Studies on 4AP derivatives including 3,4-diaminopyridine, 4-aminopyridine-3-methanol and others demonstrate that small substituents in the 3-position do not significantly impair binding to K^+^ channels^22,31–35^. Based on this, several 4AP derivatives labeled with carbon-11, namely [^11^C]3-trifluoromethyl-4AP^31^, [^11^C]3-methoxy-4AP^32^, and [^11^C]3-methyl-4AP^33^, have also been investigated (Fig. 1A). Although some of these derivatives possess advantages such as higher binding affinity and specific binding compared to [^18^F]3F4AP^31^, fluorine-18 labeled tracers are generally preferred due to their longer half-life (110 min vs*.* 20.3 min)^34^. Furthermore, [^18^F]3F4AP displays excellent characteristics for PET imaging including a fast entry and washout from the brain mediated by a p*K*_a_ value close to physiological (p*K*_a_ = 7.65) and a positive logD (logD = 0.41)^35^. In addition, recent studies have shown that 3-methyl-4AP has good binding affinity towards K^+^ channels, and thus, we hypothesized that a trisubstituted derivative of 3F4AP with a methyl group at the 5-position, 5Me3F4AP, would retain its ability to block K_v_ channels and have adequate brain permeability while exhibiting improved stability against metabolism. Herein, we characterized the pharmacological and biophysical properties of 5Me3F4AP and evaluated its in vitro metabolic stability towards CYP2E1 to investigate its potential as a PET tracer candidate for imaging demyelination.

## Results

### Basicity, lipophilicity and membrane permeability of 5Me3F4AP

Measurements of these pharmacological parameters of 5Me3F4AP were taken and compared to those of its predecessors (Fig. 1B). As indicated in left bar graph, 5Me3F4AP demonstrates comparable basicity in comparison to 3F4AP (7.46 ± 0.01 vs*.* 7.37 ± 0.07). Both compounds have p*K*_a_ values that are close to the physiological pH, indicating their coexistence in both protonated and neutral forms under physiological conditions. In contrast, 4AP and 3Me4AP display greater basicity (p*K*_a_ values above 9), indicating that the protonated form is predominant at physiological pH.

In terms of lipophilicity, 5Me3F4AP shows an octanol/water partition coefficient value at pH 7.4 of 0.664 ± 0.005 (Fig. 1B, middle), which is higher than that of 3F4AP (logD = 0.414 ± 0.002). This result indicates that both compounds preferentially partition into the octanol layer, potentially facilitating faster permeation through a lipophilic membrane like the BBB via passive diffusion. Conversely, 4AP and 3Me4AP exhibit a preference for partitioning in the water layer (logD_4AP_ =  − 1.478 ± 0.014, logD_3Me4AP_ =  − 1.232 ± 0.008), suggesting slower permeation rates. This trend was further validated through a parallel artificial membrane permeability assay, which demonstrated that 5Me3F4AP permeates approximately three times faster than 3F4AP (Fig. 1B, right).

As it may be expected, the compounds with lower p*K*_a_ exhibited higher logD and higher membrane permeability. If generalizable, this observation could lead to development of predictive models for 4AP derivatives and other basic compounds under physiological pH conditions^36^.

### Affinity towards K^+^ channels

**Figure 2:**
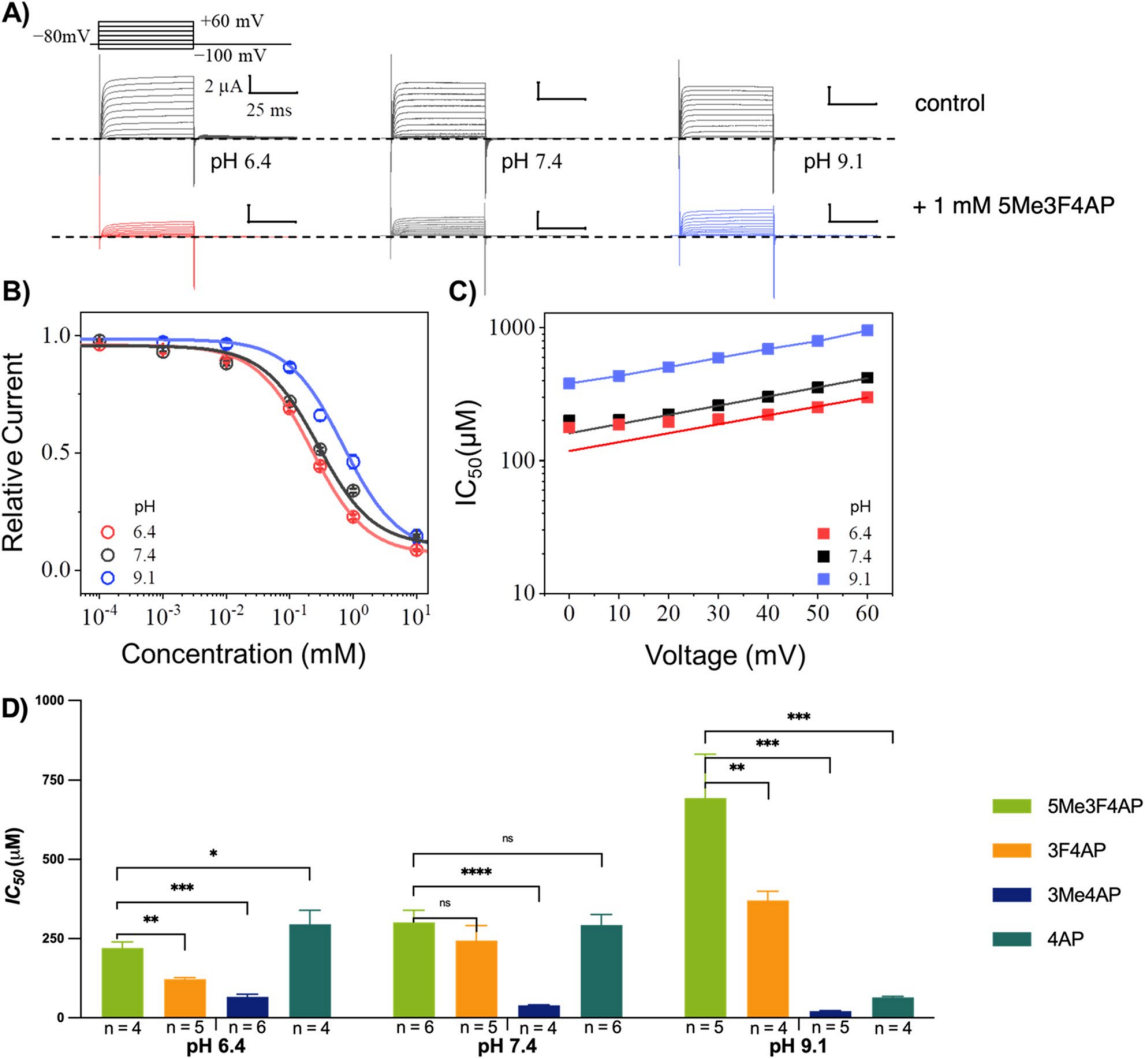
Pharmacological and biophysical characterization of 5Me3F4AP upon Shaker K_V_ ion channel. (**A**) Representative recordings at pH values of 6.4, 7.4 and 9.1 elicited from three different oocytes expressing the Shaker channel before (upper, black) and after (lower, colored) the blockage with 1 mM of 5Me3F4AP. Currents were recorded as the response to voltage stimulus protocol that consisted of 50 ms depolarization steps from − 100 to 60 mV in increments of 10 mV (top left). Dashed line represents the zero current value. Horizontal and vertical bars of 25 ms and 2 μA represent the time and current scale for all recordings. (**B**) Relative current vs. concentration of 5Me3F4AP curves assessed at 40 mV. Continuous lines represent the fits with the Hill equation. A Hill parameter of ℎ ≈ 1 was obtained during the fitting of the data for all experimental conditions. (**C**) IC_50_ vs*.* voltage curves at different pH values. Continuous lines represent the fitting to a one step binding model (Eq. 1). (**D**) Comparison of the IC_50_ at 40 mV calculated using the Hill equation for 5Me3F4AP and related compounds at different pH values. *P ≤ 0.05; **P ≤ 0.01, ***P ≤ 0.001, ****P ≤ 0.0001.

The blocking potency at different pH conditions (6.4, 7.4 and 9.1) and voltages (− 100 to 60 mV) of 5Me3F4AP was evaluated by measuring the K^+^ currents generated by Shaker voltage-gated potassium channel from *D. melanogaster* heterologously expressed in *Xenopus laevis* oocytes (Fig. 2). Specifically, Fig. 2A shows three representative recordings elicited as a response to the voltage stimulus before and after the application of 1 mM of 5Me3F4AP under three extracellular pH conditions. From these recordings, it is clear that 1 mM 5Me3F4AP efficiently blocks the channels. Figure 2B shows the dose–response plot (relative K^+^ currents at 40 mV vs. 5Me3F4AP concentration) and the fitting of the experimental values to the Hill equation to calculate IC_50_. This plot shows that blocking is dose and pH dependent with less efficient blocking at higher pH. Figure 2C shows the calculated IC_50_ values using the Hill equation at different values of pH. This plot shows that the IC_50_ of 5Me3F4AP increases with voltage and pH indicating a drop in potency. Figure 2D compares the IC_50_ values calculated at 40 mV using the Hill equation of the newly characterized 5Me3F4AP at different pHs with the IC_50_ values of the related compounds 4AP, 3F4AP and 3Me4AP. These values confirm that at pH 7.4, the blocking potency of 5Me3F4AP to K_v_ channels is similar to that of 4AP and 3F4AP previously reported^22,35^. In addition, when the pH was increased to 9.1, the IC_50_ of 5Me3F4AP (and 3F4AP) increased around three-fold indicating lower potency. Conversely, the IC_50_ from 4AP and 3Me4AP decreased by approximately four- and three-fold, respectively, indicating higher potency at higher pH. These differences in pH dependence confirm that it is the protonated form that preferentially binds to channel, since 4AP and 3Me4AP exist mostly in their protonated state at this pH range, whereas 3F4AP and 5Me3F4AP are mostly protonated at pH 6.4 and predominantly neutral at pH 9.1. In addition, we used the equation used by Hermann to analyze the voltage-dependence of 4AP^37^ to determine the IC_50(at V=0 mV)_ and the electric fraction (δ), which represents the fraction of the electric field that the compound must cross through the channel pore to reach its binding site (Table 1). A δ value of $\hspace{0.17em}\sim \hspace{0.17em}$0.4 was obtained for 5Me3F4AP; this value is consistent with the δ values of 4AP, 3F4AP and 3Me4AP previously reported^35^, and it suggests that this novel blocker binds at the same site within the Shaker pore traversing ~ 40% of the electric field generated across the lipid bilayer. Taken together, these findings suggest that the blocking potencies of these K_V_ Shaker-related channel blockers produce a trend as follows: 3Me4AP > 3F4AP ~ 4AP ~ 5Me3F4AP.

**Table 1:** IC_50_ and δ values of 5Me3F4AP and related compounds calculated using the Hermann equation.

Compound	pH	IC_50 (at V=0)_ ± s.d. (in μM)	$\delta$ ± s.d.	n
5Me3F4AP	6.4	118 ± 17	0.39 ± 0.04	4
	7.4	161 ± 25	0.40 ± 0.02	6
	9.1	373 ± 84	0.40 ± 0.04	5
4AP	6.8	178 ± 5	0.41 ± 0.06	4
	7.4^a^	155 ± 12	0.41 ± 0.08	6
	9.1	33 ± 1	0.41 ± 0.05	4
3F4AP	6.8	65 ± 3	0.41 ± 0.03	5
	7.4^a^	122 ± 4	0.46 ± 0.10	5
	9.1	159 ± 14	0.52 ± 0.02	4
3Me4AP	6.8	44 ± 1	0.28 ± 0.10	6
	7.4^a^	21 ± 1	0.43 ± 0.10	4
	9.1	10 ± 1	0.47 ± 0.03	5

^a^Data at this pH condition has been reported previously^35^.

### Metabolic stability towards CYP2E1 of 5Me3F4AP

**Figure 3:**
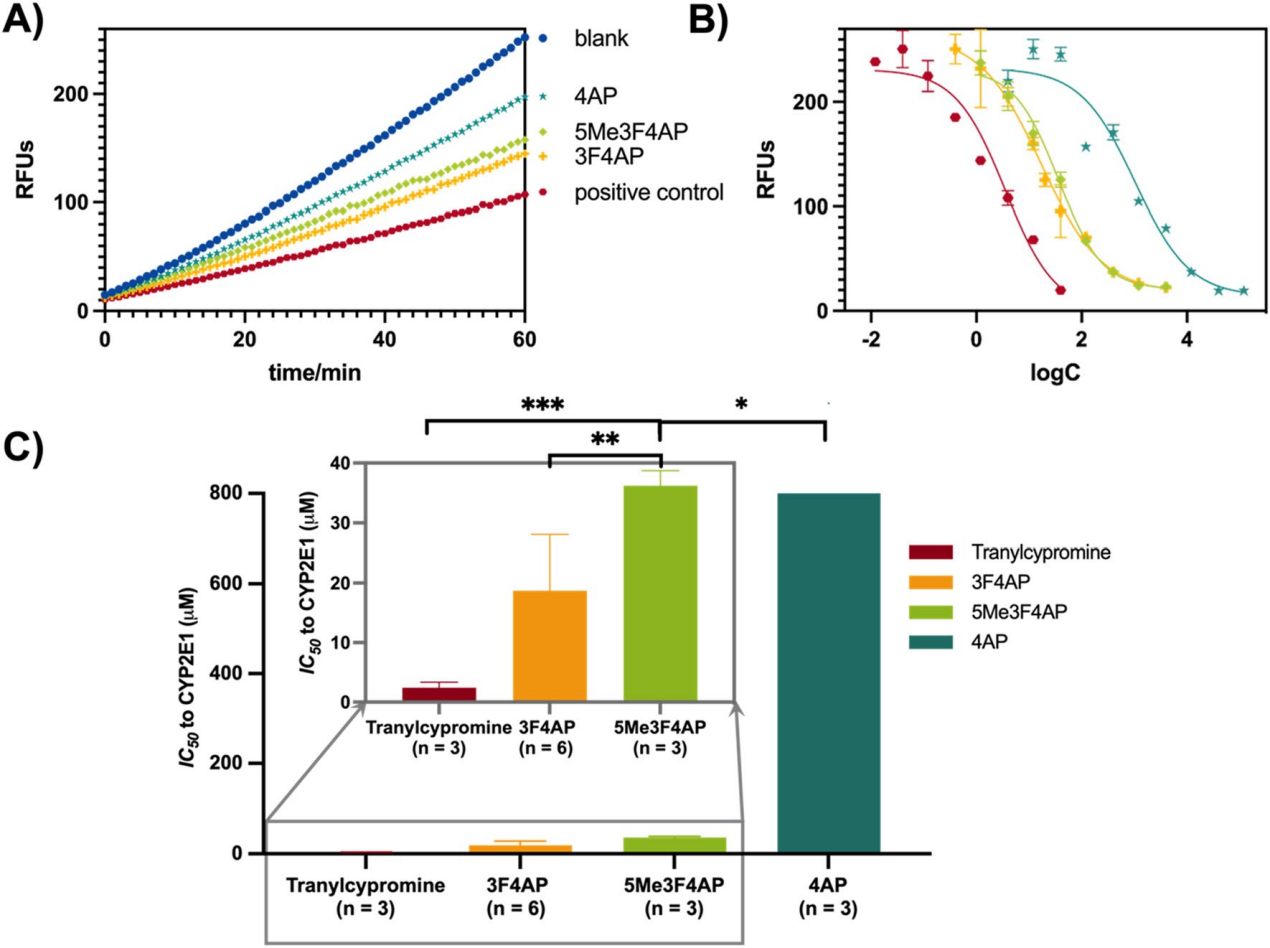
Competitive inhibition of CYP2E1. (**A**) Kinetic measurement: 60 min kinetic measurement of 4AP (cyan), 5Me3F4AP (green), 3F4AP (yellow), and tranylcypromine (red, positive control). (**B**) IC_50_ fit curves for CYP2E1 inhibition for each compound. (**C**) IC_50_ values bar graph for each compound and zoomed-in view of the bar graph focusing on tranylcypromine, 3F4AP, and 5Me3F4AP. *P ≤ 0.05; **P ≤ 0.01, ***P ≤ 0.001, ****P ≤ 0.0001.

To estimate the metabolic stability of 5Me3F4AP towards CYP2E1, we conducted an in vitro investigation utilizing a competitive inhibition assay. The protocol used for this study followed a previously established method^38^. According to the principle of this assay outlined in the method section, compounds that are good substrates of CYP2E1 result in greater reduction in the rate of formation of a fluorescent reporter than compounds that are poor substrates. In this study, we measured the reaction rates without competitor (blank) as well as in the presence of tranylcypromine (positive control), 4AP, 3F4AP and 5Me3F4AP. As illustrated in Fig. 3, the addition of tranylcypromine, a widely recognized potent substrate of CYP2E1, resulted in the most pronounced reduction in fluorogenic emission when compared to the reaction conducted without any addition of enzyme substrates (red vs. blue lines). In comparison, 4AP exhibited a minor reduction in fluorogenic emission (cyan vs*.* blue lines) indicating that it is a poor substrate of CYP2E1. 3F4AP demonstrated a substantial decrease in rate (yellow vs*.* blue lines) indicating 3F4AP is a good substrate of CYP2E1, undergoing metabolism at a much faster rate than 4AP. In comparison, 5Me3F4AP demonstrated a reaction rate between 4AP and 3F4AP, bearing a higher resemblance to 3F4AP (Fig. 3A**,** green vs*.* cyan and yellow lines). To further quantify the inhibition potency of 5Me3F4AP towards CYP2E1, we measured the CYP2E1-mediated reaction rate in the presence of varying concentrations of 5Me3F4AP and 3F4AP and performed the dose–response fitting (Fig. 3B). We also compared these results with our previous results for 4AP, 3F4AP and the positive control tranylcypromine^30^. This analysis showed that 5Me3F4AP has an IC_50_ about two times higher than 3F4AP and about 23 times lower than 4AP (Fig. 3C), indicating that it is a weaker competitive inhibitor of CYP2E1 than 3F4AP, bus stronger than 4AP. Given the previously stablished role of CYP2E1 in the in vivo metabolism of this family of compounds and the direct correlation between inhibitory potency and CYP2E1 affinity for the substrate, this finding suggests that 5Me3F4AP may be metabolized slower than 3F4AP but not as slow as 4AP.

## Discussion

This study identified 5Me3F4AP as novel trisubstituted K^+^ channel blocker with potential application for PET and examined critical pharmacological properties including lipophilicity, basicity, membrane permeability, in vitro target binding affinity and in vitro metabolic stability. While multiple disubstituted 4-aminopyridine derivatives are known to efficiently block K^+^ channels^22,35,37,39–41^, to our knowledge this is the first example of a trisubstituted derivative that can efficiently block K^+^ channels.

In comparison to its predecessor 3F4AP, 5Me3F4AP was found to have comparable basicity (p*K*_a_ 7.46 vs*.* 7.37, P-value = 0.08) and higher lipophilicity (logD of 0.66 vs*.* 0.41, P-value < 0.0001). The lower p*K*_a_ values of 3F4AP and 5Me3F4AP compared to 4AP and 3Me4AP (p*K*_a_ 9.19 and 9.82) indicate that these compounds exist both in the neutral and protonated forms at physiological pH. The protonated form readily binds to the active sites of potassium channels^37^, facilitating its pharmacological action. While the neutral form, which exhibits higher lipophilicity, can effectively penetrate the BBB through passive diffusion, enabling entry into the brain. Consequently, it is anticipated that both 3F4AP and 5Me3F4AP will have higher BBB permeability than 4AP. Furthermore, since 5Me3F4AP was found to permeate an artificial brain membrane approximately three times faster than 3F4AP it is expected that 5Me3F4AP will have higher brain uptake than 3F4AP, potentially being a better PET tracer.

Regarding blocking potency, it was found that at pH 7.4 the potency of 5Me3F4AP is very similar to that of 4AP and 3F4AP. At higher pH the potency of 3F4AP and 5Me3F4AP dropped significantly but not that of 4AP supporting that only the protonated form is able to block the channel^15^ since 3F4AP and 5Me3F4AP exist predominantly in the neutral form at basic pH. These findings suggest that 5Me3F4AP will bind to demyelinated lesions with similar sensitivity as 3F4AP. These findings suggest that 5Me3F4AP will bind to demyelinated lesions with similar sensitivity as 3F4AP. Nevertheless, the blocking potency measurements were made using the *D. melanogaster* Shaker channel and should be confirmed in human channels. The Shaker channel was used because it is easily accessible and it is an ortholog of the human channels K_v_1.1, K_v_1.2, K_v_1.3 and K_v_1.4. In addition, the Shaker channel offers the opportunity to compare our results against previous results of 4AP derivatives obtained using this channel^22,35^.

In terms of metabolic stability, 5Me3F4AP was found to undergo CYP2E1-mediated oxidation about two times slower than 3F4AP (Fig. 3A, green vs*.* yellow lines), which was further confirmed by calculating the IC_50_ (35.9 vs*.* 17.0). Although the oxidation rate of 5Me3F4AP was still significantly faster than 4AP (Fig. 3A, green vs*.* cyan lines), this reduction in the rate of oxidation suggests that 5Me3F4AP will have greater in vivo stability than 3F4AP. A limitation of this study is that we did not test other possible metabolic enzymes, which may play a role in vivo. Nevertheless, it is reasonable to test CYP2E1 given that prior studies strongly suggest this enzyme is primarily responsible for the metabolism of 3F4AP and 4AP^27,30^. Furthermore, the high in vivo stability of 4AP (~ 70% parent fraction in plasma 24 h post oral administration^42^) compared to 3F4AP (< 50% parent fraction in plasma 60 min post intravenous administration^27^) is consistent with the measured CYP2E1 oxidation rates in vitro for these compounds^30^.

In sum, the favorable in vitro characteristics of 5Me3F4AP position it as an intriguing candidate worthy of further investigation as a potential alternative to [^18^F]3F4AP for PET imaging. Nevertheless, since in vitro behavior may not accurately predict in vivo behavior, future efforts should aim to develop the radiolabeled form of this compound, testing its in vivo stability of the compound and evaluating its imaging performance. To synthesized radiolabeled [^18^F]5Me3F4AP, one may consider using similar methods to the ones previously developed to synthesize [^18^F]3F4AP such as ^18^F/^19^F exchange of a fluorinated electron-deficient pyridine N-oxide derivative followed by palladium-mediated reduction^24^ or denitroradiofluorination of a 4-methylester pyridine derivative followed by conversion of the ester group to an amino group through rearrangement^25^. In order to test the in vivo stability of [^18^F]5Me3F4AP, one can consider collecting blood at certain times post injection and analyzing for radiometabolites using a radioHPLC^26,30^. Finally, to assess the imaging properties of the tracer, one could consider performing PET imaging studies in rodent models of demyelination and comparing the binding to the lesioned areas with that of [^18^F]3F4AP^22^.

## Methods

### Animal studies compliance

Female adult frogs (*Xenopus laevis*) (acquired from Aquanimals SA de CV, Querétaro, México) were used to harvest oocytes for the studies. The animals were not euthanized as part of this study. All procedures involving frogs were performed in accordance with the ARRIVE guidelines and with the approval of the Comité Institucional del Cuidado y Uso de Animales en el Laboratorio at the University of Guadalajara (protocol: CUCEI/CINV/CICUAL-03/2023). All methods were carried out in accordance with relevant guidelines and regulations.

### Materials

5Me3F4AP was purchased from Ambeed Inc. (Arlington Hts, IL, USA). Characterization data were provided by the vendor (see spectroscopy in SI). 1H NMR (400 MHz, dmso-d_6_) δ 7.95 (d, *J* = 2.9 Hz, 1H), 7.78(s, 1H), 5.91 (s, 2H), 2.05(s, 3H); melting point: 90.9–93.8 °C.

All other chemical compounds used for this study were purchased from Sigma–Aldrich Merck (Merck KGaA, Darmstadt, Germany) or as otherwise indicated.

### Partition coefficient determination

The octanol–water partition coefficient (logD) at pH 7.4 was determined according to our previous reported protocol^35^. Briefly, PBS (900 μL), 1-octanol (900 μL), and a 10 mg/ mL aqueous solution of each compound (2 μL) were added to a 2 mL HPLC vial. The compounds were partitioned between the layers via vortexing and centrifuged at 1000* g* for 1 min to allow for phase separation. A 10 μL portion was taken from each layer (autoinjector was set up to draw volume at two different heights) and analyzed by HPLC. The relative concentration in each phase was determined by integrating the area under each peak and comparing the ratio of the areas from the octanol and aqueous layers. A calibration curve was performed to ensure that the concentrations detected were within the linear range of the detector (see Figs. S1 and S2 in Supporting Information). This procedure was repeated four times for each compound.

### ***Determination of pK***_***a***_

The p*K*_a_ was determined using titration according to our previously described protocol^35^. A 1 mg/mL solution of 5Me3F4AP was prepared, of which 5 ml was titrated with 0.01 M HCl solution beyond the equivalence point. After each incremental addition of titrant, the sample was stirred and the pH reading was taken with a pH meter. The Gran plot of the titration was analyzed to obtain the p*K*_a_ (see the plot in Supporting Information, Fig. S3). A similar protocol was used to titrate 3F4AP and 4AP, respectively (Figs. S4 and S5). The titration was repeated four times each for each compound.

### Permeability rate determination

The permeability rates of 5Me3F4AP and derivatives were determined using parallel artificial membrane permeability assay-blood–brain barrier (BBB) kit (BioAssay systems, Hayward, USA) following the manufacturer’s protocol. Initially, solutions of each test compound were prepared in DMSO at a concentration of 10 mM. These stock solutions along with the stock solutions of control compounds (high control: promazine hydrochloride, low control: diclofenac) were then diluted with PBS (pH = 7.2) to obtain the donor solutions with a final concentration of 500 μM. At the same time, 200 μM of equilibrium standards for each compound and a DMSO blank control solution were prepared.

In the experimental setup, 300 μL of PBS was added to the desired well of the acceptor plate, and 5 μL of BBB lipid solution in dodecane was added to membranes of the donor plate. Next, 200 μL of the donor solutions of each test compound and each permeability control were added to the duplicate wells of the donor plate. The donor plate was carefully placed on the acceptor plate and incubator for 18 h at room temperature. After incubation, UV absorption measurements were conducted using 100 μL of the resulting solutions from the acceptor plate and the equilibrium standards. UV absorption of the controls was measured by running a UV scan in the range of 200–500 nm. UV absorption of 5Me3F4AP and derivatives was measured using HPLC equipped with a UV detector and C18 column. The calibration curve, demonstrating the relationship between the area under the curve (AUC) in the HPLC chromatogram and concentration, is presented in the Supporting Information (Figs. S1, S2).

### Cut-open voltage clamp electrophysiology

Blocking potency of 5Me3F4AP was evaluated on the voltage-gated Shaker (homologous to mammalian K_v_1.2) ion channel expressed in *Xenopus laevis* oocytes, as previously described^35^. Briefly, each oocyte expressing the Shaker channel was voltage-clamped in a cut-open voltage clamp (COVC) station in order to elicit K^+^ currents in response to the voltage stimulus protocol, which entailed steps of 50 ms from − 100 to 60 mV in increments of 10 mV. For COVC measurements, the external recording solution was composed (in mM) of 12 KOH, 2 Ca(OH)_2_, 105 NMDG-MES (n-methyl-*d*-glucamine)-methylsufonate, 20 HEPES (4-(2-hydroxyethyl)-1-piperazineethanesulfonic acid). The internal recording solution was composed of 120 KOH, 2 EGTA (ethylene glycol-bis(β-aminoethyl ether)-N,N,N,N-tetraacetic acid), and 20 HEPES. All measurements were carried out with symmetrical (*both* internal and external) pH solutions of 6.4, 7.4 and 9.1. Each pH solution was adjusted with MES (2-(n-morpholino)ethanesulfonic acid). For measurements at pH = 9.1, HEPES was substituted by CHES (2-(cyclohexylamino)-ethanesulfonic acid). Each oocyte expressing the Shaker ion channel was voltage-clamped to record K^+^ currents, first in the absence of 5Me3F4AP, and subsequently with the addition of 5Me3F4AP, from 0.0001 to 10 mM. Relative current (I_rel_) was quantified as the ratio of the current in the absence and in the presence of the indicated concentration of 5Me3F4AP. Finally, K^+^ currents were amplified with the oocyte clamp amplifier CA-1A (Dagan Corporation, Minneapolis, MN, USA) and digitized with the USB-1604-HS-2AO Multifunction Card (Measurement Computing, Norton, MA, USA). All systems were controlled with the GpatchMC64 program (department of anesthesiology, UCLA, Los Angeles, CA, USA) via a PC. Electrophysiology recordings were sampled at 100 kHz and filtered at 10 kHz.

### Electrophysiology data analysis

Data analysis was performed as previously described^35^. Briefly, the half-maximal inhibitory concentration of 5Me3F4AP (IC_50_) was determined by fitting the I_rel_ curve to the Hill equation at each value of V and pH. A Hill coefficient (h) in the range of 0.9 < h < 1.1 was used. Voltage and pH dependence of IC_50_ was analyzed by fitting the IC_50_(V) at each pH with a simple one step binding model of 5Me3F4AP to reach its binding site (S) as previously analyzed the voltage dependence of binding of 4AP by Hermann and Gorman (Eq. 1) which allowed the determination of the fractional distance through the membrane electrical field (δ) that 5Me3F4AP has to cross to reach its binding site^43^:

1 $${\text{log}}{IC}_{50}\left(V\right)={\text{log}}{IC}_{50(V=0)}+\frac{1}{2.303}\frac{z\delta FV}{RT}$$

where IC_50(V = 0)_ is the value of IC_50_ at V = 0 mV, F is the Faraday constant, R is the gas constant, T is the room temperature, and z is the apparent charge.

Mean values of data ± standard deviation (s.d.) are given or plotted and the number of experiments is denoted by n. Upper and lower limits of the 95% of confidence interval (CI_95_) are denoted as ${10}^{\left({{\text{log}}IC}_{50}+s.d\right)}$ and ${10}^{\left({{\text{log}}IC}_{50}-s.d\right)}$, respectively.

### CYP2E1-mediated metabolic stability assessment

The relative metabolic stability towards CYP2E1 was assessed with the competitive CYP2E1 inhibition assay utilizing the life technologies^™^ vivid^®^ CYP2E1 screening kit, as described in previous studies^44^. In this assay, fluorescence emitted by the metabolic product of a specific CYP2E1 substrate included in the kit, was measured in the absence and presence of substrate competitors. Consequently, the highest fluorescence values were obtained from the blank experiments, lacking any competitors. As the concentration of competitors increased, or more potent competitors were introduced, the fluorogenic emission decreased accordingly.

Specifically, 40 µL of 2.5× (final concentration 25 µM) solution of test compounds (4AP, 3F4AP, 5Me3F4AP, and positive control, i.e., tranylcypromine) in 1× vivid^®^ CYP2E1 reaction buffer was added to desired wells of a falcon black/clear 384-well plate in three replicates. Afterwards, 50 µL master pre-mix 2× (40 nM) CYP2E1 BACULOSOMES^®^ and 2× (0.6 units/mL) vivid^®^ regeneration system in 1× reaction buffer) was added to each well. The plate was incubated for 10 min at room temperature to allow the compounds to interact with the CYP2E1 in the absence of enzyme turnover. Next, the reaction was initiated by adding 10 µL per well of 10× (100 μM) Vivid® substrate (2H-1-benzopyran-3-carbonitrile,7-(ethoxy-methoxy)-2-oxo-(9Cl)) and 10× (300 μM) Vivid® NADP^+^ mixture. Immediately (in less than 2 min), the plate was transferred into the fluorescent plate reader and fluorescence was monitored over 60 min (reads in 1 min intervals) at 415 nm as excitation wavelength and 460 nm as emission wavelength. The obtained reads were plotted using GraphPad Prism 9.

### ***Determination of the IC***_***50***_*** of 5Me3F4AP to CYP2E1***

A similar vivid^®^ CYP2E1 assay was conducted as described above. Instead of testing a single concentration of 5Me3F4AP (final concentration 15 µM), a series of concentrations (4.0 mM, 1.2 mM, 400 µM, 120 µM, 40 µM, 12 µM, 4.0 µM, 1.2 µM) were tested with three replicates for each concentration. The plate fluorescence was monitored over 60 min (reads in 1 min intervals) at 415 nm as excitation wavelength and 460 nm as emission wavelength. The reads at 60 min (recalculated by the linear trend line equation) of each concentration were used and fitted with GraphPad Prism9 dose-response-inhibition (concentration is log) curve fitting with weighting method (weighted by 1/Y^2^) to calculate the IC_50_ values. A similar procedure was used for determining the IC_50_ of 3F4AP.

### Supplementary Information

Supplementary Figures.

## Data Availability

The authors declare that all the data supporting the findings of this study are contained within the paper.
