# Supplementary material for: Chemical and biophysical characterization of novel potassium channel blocker 3-fluoro-5-methylpyridin-4-amine
Source: Sci Rep. 2024 May 15;14:11105. doi: 10.1038/s41598-024-61465-w (PMC11096398; doi:10.1038/s41598-024-61465-w)
Supplement: Supplementary file 1 — Supplementary Figures. [file 41598_2024_61465_MOESM1_ESM.docx]

**Supporting Information**

for

**Chemical and biophysical characterization of novel potassium channel blocker 3‑fluoro‑5‑methylpyridin‑4‑amine**

Yang Sun^1,§^, Sofía Rodríguez-Rangel^2,§^, Lauren L. Zhang^1^, Jorge E. Sánchez-Rodríguez^2,^* and Pedro Brugarolas^1,^*

^§^ Co-first authors / equal contribution

* Co-corresponding authors

**Affiliations**

^1^ Gordon Center for Medical Imaging, Department of Radiology, Massachusetts General Hospital and Harvard Medical School, Boston, MA, USA.

^2^ Departamento de Física, Universidad de Guadalajara, Guadalajara, Jalisco 44430, Mexico.

**Correspondence**

Pedro Brugarolas: [pbrugarolas@mgh.harvard.edu](mailto:pbrugarolas@mgh.harvard.edu)

Jorge E. Sanchez-Rodriguez: [jorge.srodriguez@academicos.udg.mx](mailto:jorge.srodriguez@academicos.udg.mx)

Contents

[**Figure S1.** **^1^H NMR** of 5Me3F4AP. 3](#_Toc164930240)

[**Figure S2.** Calibration curves of 5Me3F4AP in analytical HPLC at 254 nm (concentration in μM). 3](#_Toc164930241)

[**Figure S3.** Calibration curves of 3F4AP in analytical HPLC at 254 nm (mass in μg). 4](#_Toc164930242)

[**Figure S4.** Gran plot of 5Me3F4AP titrated with HCl. 5](#_Toc164930243)

[**Figure S5.** Gran plot of 3F4AP titrated with HCl. 5](#_Toc164930244)

[**Figure S6.** Gran plot of 4AP titrated with HCl. 6](#_Toc164930245)


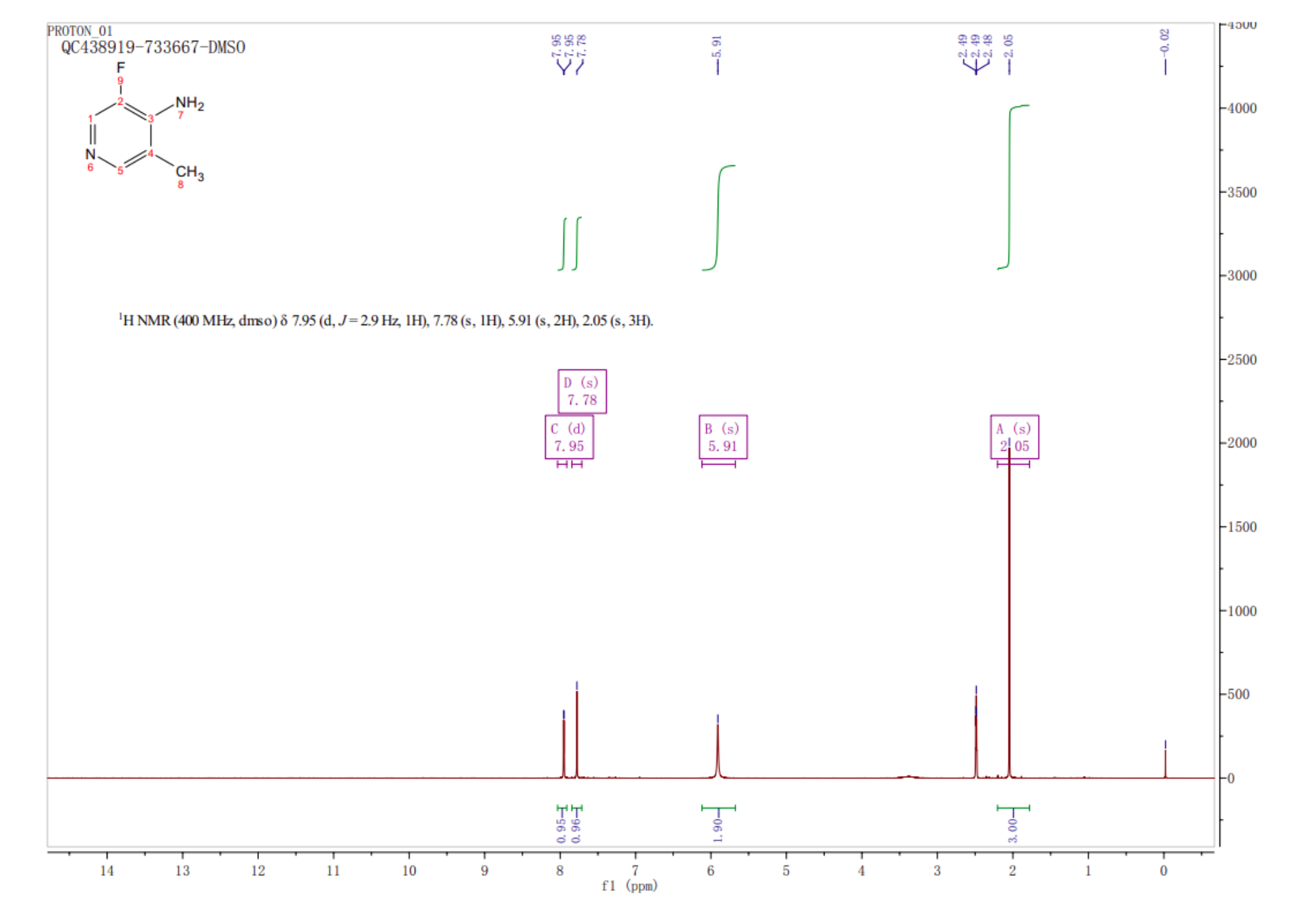


### **Figure S1.** **^1^H NMR** of 5Me3F4AP.

### **Figure S2.** Calibration curves of 5Me3F4AP in analytical HPLC at 254 nm (concentration in μM).

### **Figure S3.** Calibration curves of 3F4AP in analytical HPLC at 254 nm (mass in μg).

### **Figure S4.** Gran plot of 5Me3F4AP titrated with HCl.

### **Figure S5.** Gran plot of 3F4AP titrated with HCl.

### **Figure S6.** Gran plot of 4AP titrated with HCl.
